# Protocol for flow cytometry immunophenotyping of human antigen-specific T cells by activation-induced marker and Th1 cytokine detection

**DOI:** 10.1016/j.xpro.2024.103343

**Published:** 2024-12-19

**Authors:** Gianluca Rotta, Valentina Achille, Scott J. Bornheimer, Melanie Duenas, Marco Fernandez, Arianna Gatti, Sergio Haro Giron, Irene Martinez Rio, Marta Massanella, Diego J. Jiménez, Jorge Monserrat, Verena Pfeifer, Josefa Pichler, Barbara Prietl, Harald Sourij, Chiara R.M. Uras, Sara Vlah, Daniela Fenoglio

**Affiliations:** 1 Becton, Dickinson & Company, 20161 Milan, Italy; 2 Cytogenetics and Molecular Genetics Laboratory ASST Ovest Milanese - Legnano Hospital, 20025 Legnano, Italy; 3 Becton, Dickinson & Company, Milpitas, CA 95035, USA; 4 Becton, Dickinson & Company, 28522 Madrid, Spain; 5 Flow Cytometry Core Facility Germans Trias i Pujol Research Institute, 08916 Badalona, Spain; 6 Transfusion Center ASST Ovest Milanese - Legnano Hospital, 20025 Legnano, Italy; 7 Department of Medicine and Medical Specialties, University of Alcalá, 28801 Alcalá de Henares, Spain; 8 IrsiCaixa AIDS Research Institute, Germans Trias i Pujol Research Institute (IGTP), Can Ruti Campus, Badalona, Catalonia, Spain; 9 Center for Biomarker Research in Medicine GmbH, Graz 8010, Austria; 10 Becton, Dickinson & Company, Vienna 1200, Austria; 11 Division of Endocrinology and Diabetology, Medical University of Graz, Graz 8036, Austria; 12 Department of Experimental Medicine, Centre of Excellence for Biomedical Research, University of Genoa, 16132 Genoa, Italy; 13 Department of Internal Medicine, University of Genoa, 16132 Genoa, Italy; 14 IRCCS Ospedale Policlinico San Martino, 16132 Genoa, Italy

**Keywords:** Cell Biology, Cell culture, Cell-based Assays, Flow Cytometry, Immunology

## Abstract

Flow cytometry characterization of antigen-specific polyfunctional T cells is a valuable tool to study adaptive immunity. Here, we present a protocol for flow cytometry immunophenotyping of human antigen-specific T cells by activation-induced marker (AIM) and Th1 cytokine detection. We describe steps for preparing peripheral blood mononuclear cells (PBMCs) for stimulation followed by washing and staining PBMCs for flow cytometry. We then detail procedures for acquisition and analysis. This protocol has potential applications in the field of vaccine immunology and immuno-oncology.

For complete details on the use and execution of this protocol, please refer to Altosole et al.^1^

## Highlights

• Antigen-specific T cell detection based on intracellular staining of CD137 and CD69
• Activation-induced marker evaluation in conjunction with Th1 cytokine detection
• T cell maturation curves based on CD45RA and CCR7 are included in the panel
• Detection of antigen-specific T follicular helper cells is supported by CXCR5 staining

## Abstract (editor highlights)

Publisher’s note: Undertaking any experimental protocol requires adherence to local institutional guidelines for laboratory safety and ethics.

## Graphical abstract

**Figure:**
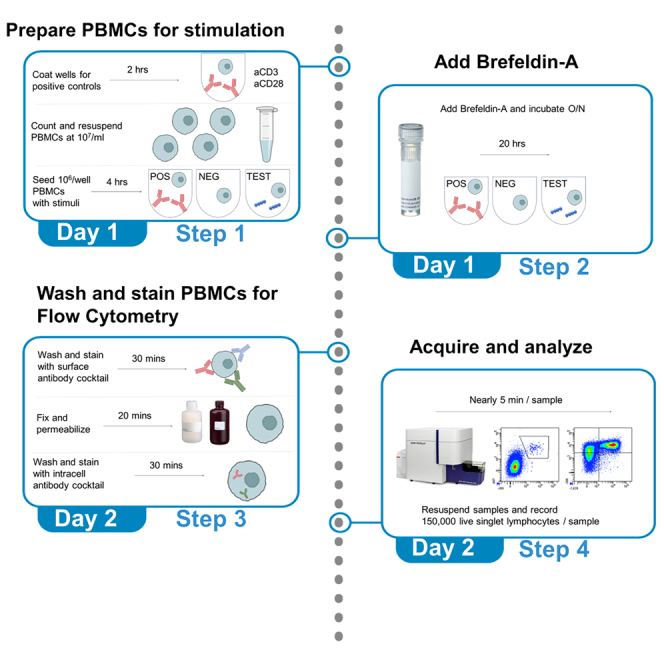

## Before you begin

The following protocol describes steps for flow cytometry detection and immunophenotyping of human antigen-specific T cells recognizing SARS-CoV-2 Spike protein. It is based on the intracellular evaluation of activation induced markers CD137 and CD69 following overnight incubation of peripheral blood mononuclear cells (PBMCs) with Spike protein peptide pool. Activation is assessed on live singlet CD3+ CD4+ and CD3+ CD8+ T lymphocytes which are also evaluated for intracellular accumulation of IFN-γ, TNF-α and IL-2 cytokines. Surface staining of CD45RA and CCR7 is used to describe the T cell maturation curve and the CXCR5 chemokine receptor, evaluated on live CD3+ CD4+ CD45RA- populations, is used to capture T helper follicular cells.

To develop the protocol, frozen PBMCs from SARS-CoV-2 reactive samples were used, being reactivity induced by vaccination, natural infection, or both; but fresh PBMCs may also be used. Each sample is divided into three aliquots of 10^6^ PBMCs/each as positive control, negative control, and test. Incubation with Spike peptide pools is done overnight taking 24 h, and protein transport inhibitor Brefeldin A is added after the first 4 h. Following incubation, cells are washed and stained for surface markers and viability, being then fixed, permeabilized and stained for intracellular markers and cytokines. Flow cytometry acquisition of 150,000 live singlet lymphocytes / sample can be done the same day of the staining or the following day.

A major challenge in optimizing this protocol was identifying markers and conditions suitable for simultaneous AIM testing and intracellular Th1 cytokine detection for the characterization of antigen specific polyfunctional T cells.

CD137 is normally evaluated in conjunction with OX40 and CD69 to detect antigen specific CD4+ and CD8+ T subsets respectively,^2^^,^^3^ and has been among the first published markers.^4^ Nevertheless, in our hands the conditions for optimal cytokine staining were not compatible with the evaluation of surface CD137. Since the literature already reported intracellular (ic) CD137 staining in conjunction with cytokine detection,^5^^,^^6^^,^^7^ we further explored if ic-OX40 and ic-CD69 could be successfully adopted in parallel. Following antigen activation, we found that ic-CD69 showed full correlation with ic-CD137 and captured the entire cytokine producing subsets on both CD4+ and CD8+ T cells. On the contrary ic-OX40 expression (expected only on CD4+ T cells) showed very poor concordance with ic- CD137 expression and with cytokine production.^1^

The possibility to stain activated CD4+ and CD8+ T cells using two markers only (ic-CD137 and ic-CD69) gave us in the panel for another marker, and CXCR5 was chosen for the detection of CD4+ Tfh cells. We did not further explore the expression of additional activation markers like CD40L and CD25. It is known that the former can be detected in the cytoplasm and used in conjunction with ic- CD137,^6^ whereas increase of CD25 expression takes longer than 24 h.^8^ Given that in our hands ic-CD137 and ic-CD69 were highly correlated, we speculated that adding further markers would have been redundant. Additionally, CD69 was favored as an early activation marker^9^ thus helping to ensure (in parallel with negative control) that the detected T cell activation following the 24 h stimulation is generated in the context of the experiment and is not pre-existing.

Finally, whereas this protocol has been set and fine-tuned using Spike peptide pools to activate SARS-CoV-2 reactive samples, proof of principle data has been generated to detect CMV reactive T cells following stimulation with CMV pp65 and CMV IE-1 peptide pools.^1^ This opens the door to application of this protocol, with changes in activating peptides, to other T cell activation settings.

Refer to the key resources table for a complete list of reagents and tools.

### Institutional permissions

All participants provided written informed consent, and this study was approved by the institutional review boards of each participating site and was conducted in accordance with ethics guidelines, including the Declaration of Helsinki.

### Preparation of Peptivator™ S, S1, S+ spike peptide pool mix

**Timing: 1 h (the day before the experiment)**

To reconstitute the lyophilized peptide pools.

1. Take the vials from −20°C and warm-up to 18°C–25°C
2. Open the rubber cap and add 200 μL of sterile distilled water to each vial using the p200 pipette.
3. Prepare 5 μL working aliquots from the stock solutions.
4. Store the working aliquots at −80°C.

**CRITICAL:** Three different Spike peptide pools are used in this protocol, named S, S1, S+. Each of them must be reconstituted and aliquoted separately avoiding cross-contamination. Sterility is fundamental: work under biosafety cabinet and use sterile materials. Clearly label all the vials for working aliquots with permanent marker. Thawed aliquots must be used immediately.

### Preparation of BD Horizon™ fixable viability stain 575V

**Timing: 1–2 h, depending on the aliquots volume (the day before the experiment)**

5. Take the vial from −80°C and warm-up to 18°C–25°C.
6. Add 340 μL of fresh cell culture-grade Dimethyl Sulfoxide (DMSO).
7. Vortex solution well until the stock dye has fully dissolved.
8. Prepare 2 μL or 5 μL working aliquots from the stock solutions.
9. Store the working aliquots at −20°C.

**CRITICAL:** Working aliquots of BD Horizon™ Fixable Viability Stain 575V (FVS) should be discarded 90 days after reconstitution with DMSO.

Note that to stain samples, working aliquots require 1:100 dilution, and 10 μL/sample of diluted solution are used. As such, a 2 μL undiluted aliquot can be used to stain 20 samples and a 5 μL aliquot can be used to stain 50 samples. Carefully decide the volume of your aliquots to balance time for aliquoting and minimize product waste. Thawed aliquots must be used immediately.

## Key resources table

**Table:** 

REAGENT or RESOURCE	SOURCE	IDENTIFIER
**Antibodies**		

BD® PerCP-Cy™5.5 mouse anti-human CD3, clone SK7. Dilution 1:7.5	BD Biosciences	332771
BD® APC-H7 mouse anti-human CD4, clone SK3. Dilution 1:17.2	BD Biosciences	641398
BD Horizon™ V500-C mouse anti-human CD8, clone SK1. Dilution 1:17.2	BD Biosciences	647457
BD Pharmingen™ PE mouse anti-human CCR7, clone 2-L1-A. Dilution 1:17.2	BD Biosciences	566741
BD Horizon™ BV786 mouse anti-human CD45RA, clone HI100. Dilution 1:17.2	BD Biosciences	563870
BD Horizon™ BV421 rat anti-human CXCR5, clone RF8B2. Dilution 1:17.2	BD Biosciences	562747
BD Pharmingen™ APC mouse anti-human CD137, clone 4B4-1. Dilution 1:7.5	BD Biosciences	550890
BD Pharmingen™ PE-Cy™7 mouse anti-human CD69, clone FN50. Dilution 1:30	BD Biosciences	557745
BD Pharmingen™ FITC mouse anti-human IFNγ, clone B27. Dilution 1:75	BD Biosciences	554700
BD Horizon™ BV711 mouse anti-human IL-2, clone 5344.111. Dilution 1:60	BD Biosciences	563946
BD Pharmingen™ Alexa Fluor™ 700 mouse anti-human TNF-α, clone MAb11. Dilution 1:240	BD Biosciences	557996
BD Pharmingen™ purified NA/LE mouse anti-human CD3, clone UCHT1. Dilution 1:1,000	BD Biosciences	555329
BD Pharmingen™ purified NA/LE mouse anti-human CD28, clone CD28.2. Dilution 1:1,000	BD Biosciences	555725

**Chemicals, peptides, and recombinant proteins**		

BD Pharmingen™ stain buffer (BSA)	BD Biosciences	554657
BD Cytofix/Cytoperm™ Plus Fixation/Permeabilization Solution Kit with BD GolgiPlug™	BD Biosciences	555028
BD Pharmingen™ Human BD Fc Block™	BD Biosciences	564220
BD Horizon™ Fixable Viability Stain 575V	BD Biosciences	565694
PepTivator™ SARS-CoV-2 Prot_S+, research grade	Miltenyi Biotec	130-127-311
PepTivator™ SARS-CoV-2 Prot_S, research grade	Miltenyi Biotec	130-126-700
PepTivator™ SARS-CoV-2 Prot_S1, research grade	Miltenyi Biotec	130-127-041
Gibco™ RPMI 1640 medium	Thermo Fisher Scientific	11875093
Gibco™ Fetal bovine serum, qualified, heat inactivated, United States	Thermo Fisher Scientific	16140071
Gibco™ Antibiotic-Antimycotic (100×)	Thermo Fisher Scientific	15240062
Gibco™ DPBS, no calcium, no magnesium	Thermo Fisher Scientific	14190144
DMSO	Merck	D8418-50ML
Distilled water	N/A	N/A

**Biological samples**		

Human PBMCs reactive to spike protein	IRCCS Ospedale Policlinico San Martino – Genoa 16132 – Italy; Legnano Hospital, Legnano 20025 Italy; Germans Trias i Pujol Research Institute, Badalona 08916 – Spain; University of Alcalá, Alcalá de Henares 28801 – Spain: Medical University of Graz, Graz 8036 – Austria	

**Software and algorithms**		

BD FACSuite™ Software	BD Biosciences	666983

**Other**		

BD® CompBeads anti-mouse Ig, κ/negative control compensation particles set	BD Biosciences	552843
BD® CompBeads anti-rat and anti-hamster Ig κ/negative control compensation particles set	BD Biosciences	552845
BD FACSLyric™ Flow Cytometer	BD Biosciences	663029
Falcon™ 96-well clear round-bottom not treated microplate, with lid, individually wrapped, sterile, 50/case	Corning Life Sciences	351177
Falcon™ 5 mL round-bottom polystyrene test tube, without cap, sterile, 125/pack, 1000/case	Corning Life Sciences	352052
Falcon™ 5 mL round-bottom PP test tube, without cap, sterile, 125/pack, 1000/case	Corning Life Sciences	352053

## Materials and equipment

**Table:** RPMI 1640 complete medium

Reagent	Final concentration	Amount
RPMI 1640	–	500 mL
Gibco™ Fetal Bovine Serum, qualified, heat inactivated, United States	10%	56.1 mL
Gibco™ Antibiotic-Antimycotic (100×)	1% (v/v)	5.6 mL
**Total**	**N/A**	**561.7 mL**

RPMI 1640 Complete Medium is required on day 1 to resuspend and seed the cells.

Keep Sterile - Store at +4°C for up to 3 months.

***Alternatives:*** Flow Cytometer. Any platform equipped with Blue, Red and Violet Laser, optionally equipped with Yellow Green Laser and or UV laser, compatible with the dyes used in this protocol.

The following platforms have been used with this protocol.

• BVR BD FACSCelesta™ Flow Cytometer;
• BVRYGUV BD FACSymphony™ A3 Flow Cytometer;
• BVRYGUV BD FACSymphony™ A5SE Flow Cytometer;
• BVRYGUV BD LSRFortessa™ Flow Cytometer;
• BVRYG BD LSR II™ Flow Cytometer;
• BVRYG BD FACSAria™ II Cell Sorter.

These platforms are equipped with BD FACSDiva™ Software.

**Table:** Peptivator™ S, S1, S+ spike peptide pool mix (example for 10 samples)

Reagent	Volume/sample	Final amount
Peptivator™ S	2 μL /sample	22 μL (2 μL overage)
Peptivator™ S1	2 μL /sample	22 μL (2 μL overage)
Peptivator™ S+	2 μL /sample	22 μL (2 μL overage)
**Total**	**6 μL /sample**	**66 μL**

Peptivator™ S, S1, S+ Mix is required on day 1 immediately before seeding cells. Prepare it from working aliquots stored at −80°C and discard the remnant volume which is not used in the experiment.

### Instrument setting

In this session useful references are provided for instrument setting and compensation calculation.

Working on the BD FACSLyric™ Flow Cytometer does not require any change in the default photomultiplier amplification provided with the embedded “LYSE WASH” setting. For any other instrument equipped with photomultiplier tubes we do recommend the “Voltage titration” protocol aimed at determining the optimal amplification for each fluorescent channel. Details can be found at https://www.bdbiosciences.com/content/dam/bdb/marketing-documents/BD-FACSDiva-Initial-PMT-Voltages.pdf.

For the compensation procedure the following protocol must be used https://www.bdbiosciences.com/en-us/products/reagents/flow-cytometry-reagents/research-reagents/compensation-beads.

Fixable Viability dye signal can be left uncompensated as all positive cells are excluded from the gating strategy.

## Step-by-step method details

### Coating of wells dedicated to positive controls with purified anti CD3 and anti CD28 monoclonal antibodies

**Timing: 2 h 30 min or overnight**

This section describes the preparation of the culture plates which is required by positive control samples incubated in wells coated with CD3 and CD28 antibodies.

Evaluation of antigen specific T cells requires a positive control for each of the assessed samples to demonstrate its capacity of upregulating activation markers following stimulation with CD3 and CD28 monoclonal antibodies (mAbs). Positive controls are created by incubating PBMCs in CD3 and CD28 coated wells. Coating can be done for 2 h at +37°C or Over Night at +4°C. Carefully plan the coating time to avoid delay in the experimental workflow.

**CRITICAL:** This step requires sterility, wear gloves and work under sterile hood.

***Note:*** CD3 and CD28 mAbs for positive control have a bulk concentration of 1 mg/mL. Working concentration for both is 1 μg/mL: dilute the stock 1:1000 in DPBS to have 1× solution.

1. Dispense 250 μL/well.
2. Seal the plate and incubate either for 2 h at +37°C or Overnight at +4°C.
3. After incubation, empty the wells by plate inversion (maintain sterility).
4. Wash the wells three times by adding 250 μL/well of DPBS.
5. If cells are not ready for the experiment do not let wells dry, maintain DPBS in the wells.

### PBMC stimulation (day 1)

**Timing: 30 min dispensing stimuli and seeding the cells + 4 h incubation without Brefeldin A + 20 h incubation with Brefeldin A**

This section describes how to prepare the appropriate stimuli and seed the cells. PBMCs must be thawed immediately before use according to the preferred laboratory protocol and resuspended at a concentration of 10^7^/mL in RPMI 1640 complete medium.

The evaluation of each sample requires a negative control without stimulation, a positive control which is seeded in wells coated with CD3 and CD28 mAbs and a test sample which is incubated in the presence of Peptivator™ S, S1, S+ Spike peptide pool Mix.

**CRITICAL:** Seeding a constant number of 10^6^ cells/wells is crucial to maintain a fixed ratio between cells and stimuli.

6. Remove any residual DPBS from wells coated with CD3 and CD28 mAbs dedicated positive controls.
7. Add to each test sample well 6 μL of Peptivator™ S, S1, S+ Spike peptide pool Mix. Keep test wells far from negative control wells (See troubleshooting problem 1).
8. Add to each negative control well 6 μL of DI water.
9. Dispense 100 μL of cell solution in each well (10^6^ cells). Mix gently and carefully with multichannel pipette.
10. Place into incubator for 4 h.
11. During this incubation time prepare Brefeldin A (Golgi Plug): dilute 1 μL in 100 μL of RPMI 1640 Complete Medium to have 10× solution.
12. After 4 h dispense 10 μL of Brefeldin A (Golgi Plug) diluted solution to each well and pipette gently.
13. Incubate 20 h overnight at 37°C.

**CRITICAL:** Do not use CD28 co-stimulation to enhance T cell stimulation.

***Note:*** Following the protocol development,^1^ we further tested the effect of using or not using CD28 co-stimulation (1 μg/mL) on 26 negative controls and Spike stimulated samples acquired in five different laboratories. To read out the effect, we calculated for each stimulated-unstimulated paired samples the Stimulation Index (S.I.) using the formula reported below.

$$CD4+S.I.=\frac{\%\;of\;{\left(\text{AIM}+\text{CD}4+\text{Tcell}\right)}_{\text{stimulated}\;}}{\%\;of\;{\left(\text{AIM}+\text{CD}4+\text{Tcell}\right)}_{\text{unstimulated}}}$$

CD8+ S.I. is calculated with analogous formula.

Percentages are calculated out of total CD4+ or out of total CD8+.

AIM+ indicates T cells being icCD137bright and ic-CD69+

**Figure 1:**
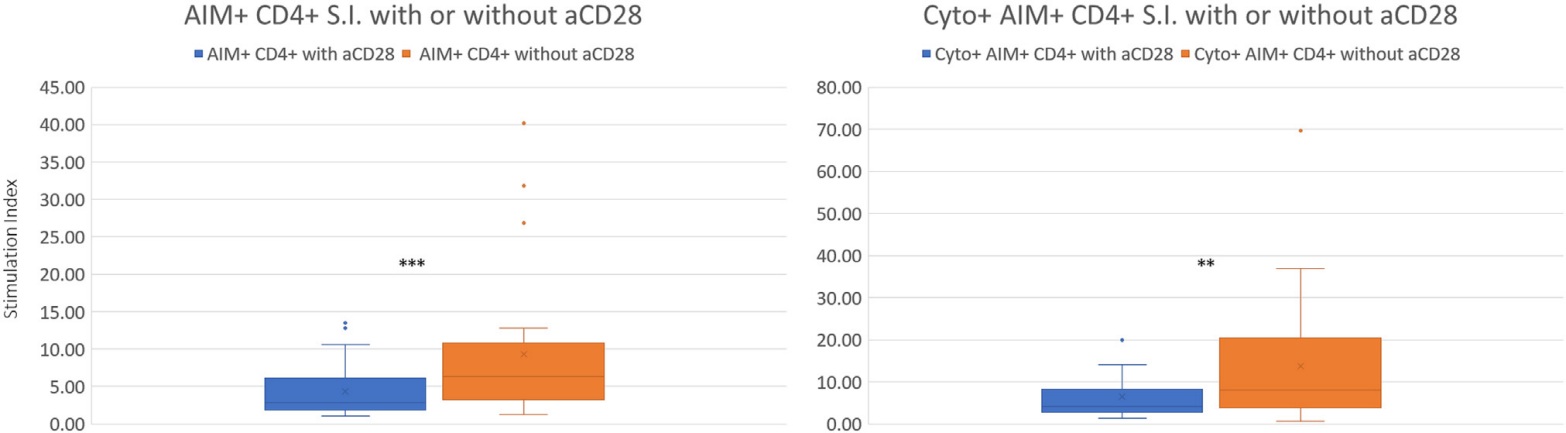
CD4+ stimulation index with or without CD28 co-stimulation 26 samples were stimulated with Peptivator™ S, S1, S+ Spike peptide pool Mix in the presence or absence of CD28 used as co-stimulus. Unstimulated samples were treated accordingly with or without CD28. Multicolor flow cytometry revealed a significantly higher Stimulation Index when samples were not co-stimulated with CD28. The pattern was conserved for total activated (AIM+, left panel) and for cytokine producing (Cyto+AIM+, right panel) CD4+ T cells (∗ = *p* < 0.05; ∗∗ = *p* < 0.005; ∗∗∗ = *p* < 0.0005).

**Figure 2:**
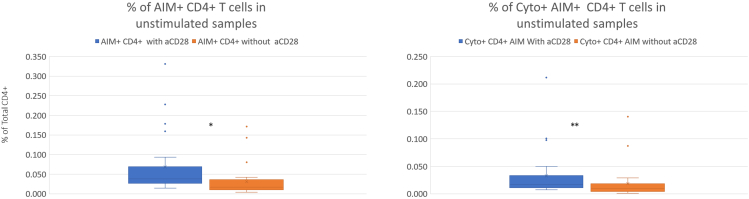
CD4+ background activation CD4+ T cell background activation was lower in the absence of CD28 co-stimulation, for both total activated CD4+ T cells (AIM+, left panel) and for the cytokine-producing subset (Cyto+ AIM+, right panel) (∗ = *p* < 0.05; ∗∗ = *p* < 0.005; ∗∗∗ = *p* < 0.0005).

Samples incubated without CD28 mAb revealed a significantly higher mean CD4+ S.I. (9.4 vs. 4.3; *p*-Value = 0.001) and mean cytokine-producing CD4+ S.I. (13.6 vs. 6.4; *p*-Value = 0.002) (Figure 1), which was driven by a 54% reduction of background activation in unstimulated samples for the total activated CD4+ subset and by a 43% reduction of background activation for the cytokine producing CD4+ subset (Figure 2). In parallel with decreased background activation in unstimulated samples, specific activation was also lower in stimulated samples when CD28 was not used (reduction of activation was 18% and 16% for CD4+ and for cytokine-producing CD4+ respectively). Notably the effect of background reduction was dominant thus leading to the significant increase of S.I. in the absence of CD28 co-stimulation. No relevant variations were reported for the CD8+ compartment.

**Table 1:** Blocking solution (example for 10 samples)

Reagent	Volume / sample	Final amount
FC Block	1 μL /sample	12 μL (2 μL overage)
Brilliant Stain Buffer	25 μL /sample	300 μL (50 μL overage)
**Total**	**26 μL /sample**	**312 μL**

**Table 2:** Reagent cocktail for PBMC surface staining (example for 10 samples)

Reagent	Volume / sample	Final amount
Brilliant Stain Buffer	25 μL /sample	250 μL
CD4 APC-H7	5 μL /sample	50 μL
CD8 V500-C	5 μL /sample	50 μL
CD45RA BV786	5 μL /sample	50 μL
CCR7 PE	5 μL /sample	50 μL
CXCR5 BV421	5 μL /sample	50 μL
FVS575V	10 μL /sample	100 μL
**Total**	**54 μL /sample (90% of theoretical volume)**	**600 μL**

**Figure 3:**
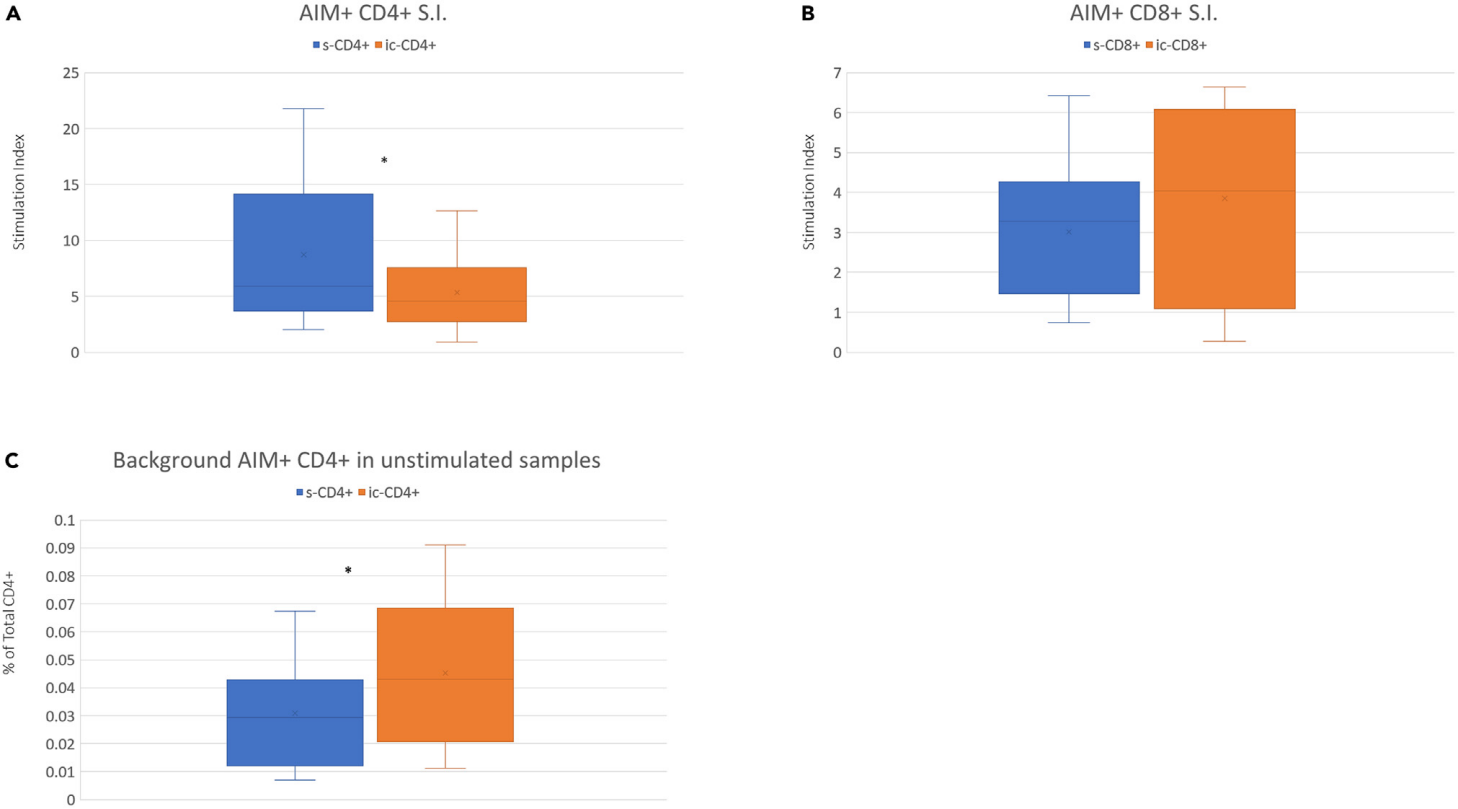
CD4+ and CD8+ T cell stimulation index with surface and intracellular staining 9 samples were stimulated with Peptivator™ S, S1, S+ Spike peptide pool mix and subsequently stained with the 12-color panel using surface or intracellular CD4 and CD8 staining. Stimulation Index is calculated as previously explained. The use of intracellular CD4 and CD8 markers did not result in higher Stimulation Index (A and B), rather ic-CD4 staining resulted in higher background activation (C) thus lowering the related Stimulation Index (A) (∗ = *p* < 0.05).

**Table 3:** Reagent cocktail for PBMC intracellular staining (example for 10 samples)

Reagent	Volume / sample	Final amount
Brilliant Stain Buffer	50 μL /sample	500 μL
CD3 PerCP-Cy5.5	20 μL /sample	200 μL
CD69 PE-Cy7	5 μL /sample	50 μL
CD137 APC	20 μL /sample	200 μL
IFNγ FITC	2 μL /sample	20 μL
IL-2 BV711	2.5 μL /sample	25 μL
TNF-α Alexa Fluor® 700	0.625 μL /sample	6.25 μL
**Total**	**90 μL /sample (90% of theoretical volume)**	**1001.25 μL**

### Processing of PBMCs after stimulation (day 2)

**Timing: 2 h 30 min**

This section describes the staining and fixation protocol for surface and intracellular markers.

**CRITICAL:** Surface and intracellular antibody cocktails must be prepared immediately before use: typically, surface cocktail is prepared at the end of incubation time and intracellular cocktail is prepared during cell fixation time.

**CRITICAL:** For antibody cocktail preparation, Brilliant Stain Buffer must be always dispensed first.

**CRITICAL:** It is not required to consider an excess of volume for the antibody cocktails preparation: the protocol has been set preparing the exact volume calculated for the number of evaluated samples and then dispensing 90% of the required theoretical reagent volume (See table as example).

14. Prepare the blocking solution as indicated in Table 1 and store it at 4°C in the dark until use.
15. FVS575V: dilute stock solution 1:100 in PBS. Consider that 10 μL/well of diluted solution will be required.
16. Prepare the surface reagent cocktail as indicated in Table 2 and store it at 4°C in the dark until use.
17. At the end of incubation add 100 μL of DPBS into each well.

**CRITICAL:** Do not use BSA containing buffer for step 26 as it will interfere with FVS staining.

18. Spin down the plate at 400 g for 5 min.
19. Empty wells by inversion.
20. Dispense in each well 26 μL of Blocking Solution (Brilliant Stain Buffer + Fc-Block as per Table 1) and incubate 10 min.
21. Dispense in each well 54 μL of Surface antibody mix including FVS and Brilliant Stain Buffer and resuspend gently.
22. Incubate for 20 min at 18°C–25°C in the dark.
23. During surface staining, dilute Perm/Wash Buffer from 10× to 1× in distilled water. A volume of 300 μL/sample will be required.
24. At the end of antibody incubation, add 100 μL of Stain Buffer BSA to each well and spin down at 400 g for 5 min. Empty the wells by inversion.
25. Add 200 μL of Fixation/Permeabilization solution, pipette to resuspend the cells and incubate for 20 min at 18°C–25°C in the dark. If clogs are visible remove them by using a pipette (See troubleshooting problem 2).
26. Use this incubation time to prepare the intracellular Antibody Mix as indicated in Table 3. Spin the cocktail down at 5,000–10,000 g (according to maximum centrifuge rotation speed) for 10 min to avoid staining smears (See troubleshooting problem 3).
27. Spin down the plate at 400 g for 5 min and empty well by inversion.
28. Wash with 200 μL of Perm/Wash Buffer at working concentration and pipette to resuspend the cells.
29. Spin down at 400 g for 5 min and empty wells by inversion.
30. Add 90 μL of the reagent mix for IC staining and gently pipette.
31. Incubate 20 min at 18°C–25°C in the dark.
32. After incubation wash once with 100 μL of Perm/Wash Buffer and spin down as done in previous steps.
33. Transfer into 5 mL FACS tubes and resuspend in 400 μL of DPBS for acquisition.
34. Acquire samples using the intermediate or lowest flow rate and always run distilled water for 1 min between samples to avoid clogs. Set target acquisition at 150,000 live singlet lymphocytes (See troubleshooting problem 4).

***Note:*** Although intracellular staining is mandatory for the CD3 marker, this is not required for the CD4 and CD8 molecules. CD4+ and CD8+ intracellular vs surface (s) staining was assessed in nine donors, revealing a lower mean ic-CD4+ vs s-CD4+ S.I. (ic-CD4+ S.I. = 5.3; s-CD4+ S.I. = 8.7; p-Value = 0.036) (Figure 3). No significant differences were detected for the CD8+ subset. For the purpose of this test CD4 APC-H7 and CD8 V500-C reagents were added either to the surface or to the intracellular antibody cocktails and samples were stained in parallel after activation.

## Expected outcomes

**Figure 4:**
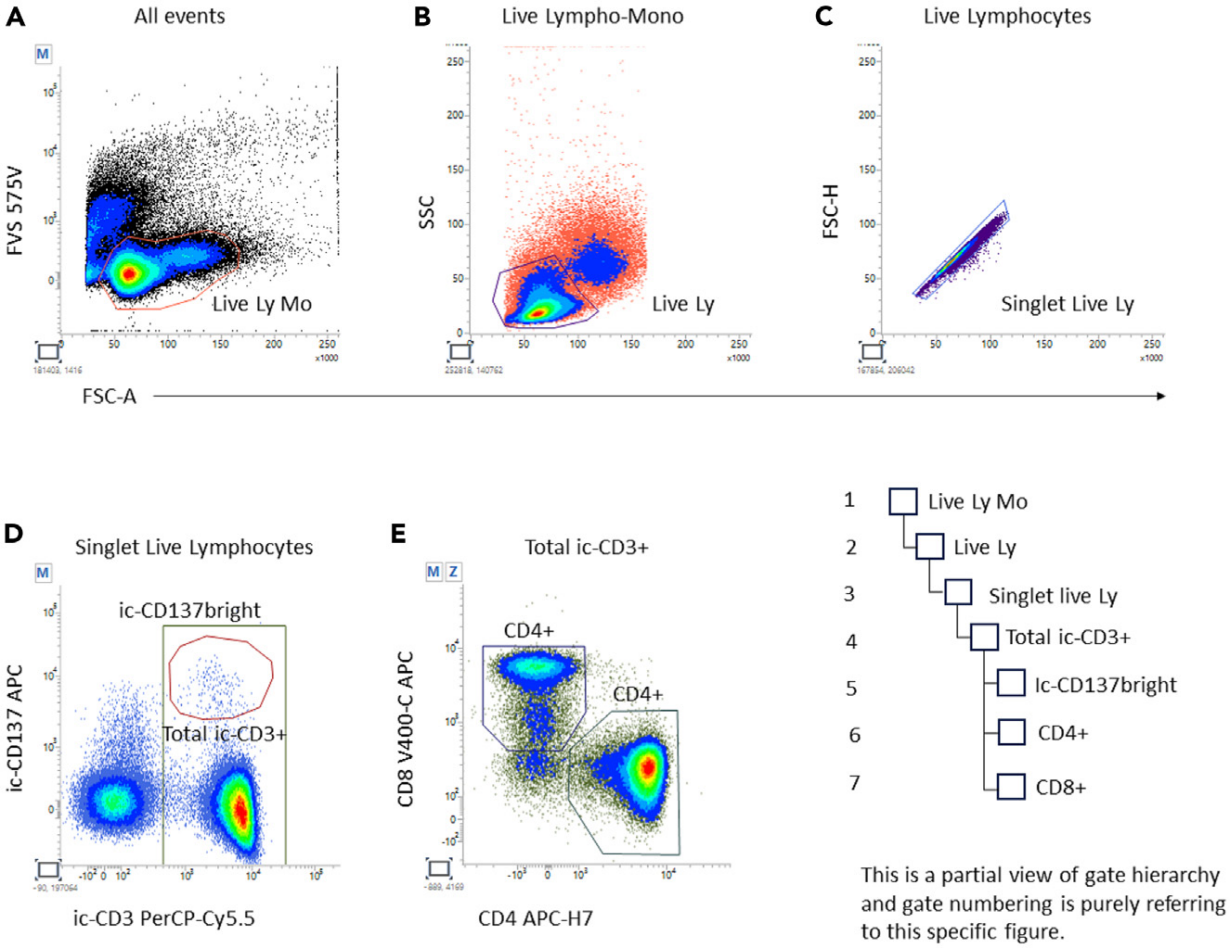
Definition of the main T cell populations of interest and related gate hierarchy Plots from (A–E) display the relevant gates around the following subsets: Live Lympho Monocytes (A), Live Lymphocytes (B), SInglet live lymphocytes (C), Total ic-CD3 and ic-CD137bright ic-CD3 (D), and CD4+ and CD8+ T cells (E).

**Figure 5:**
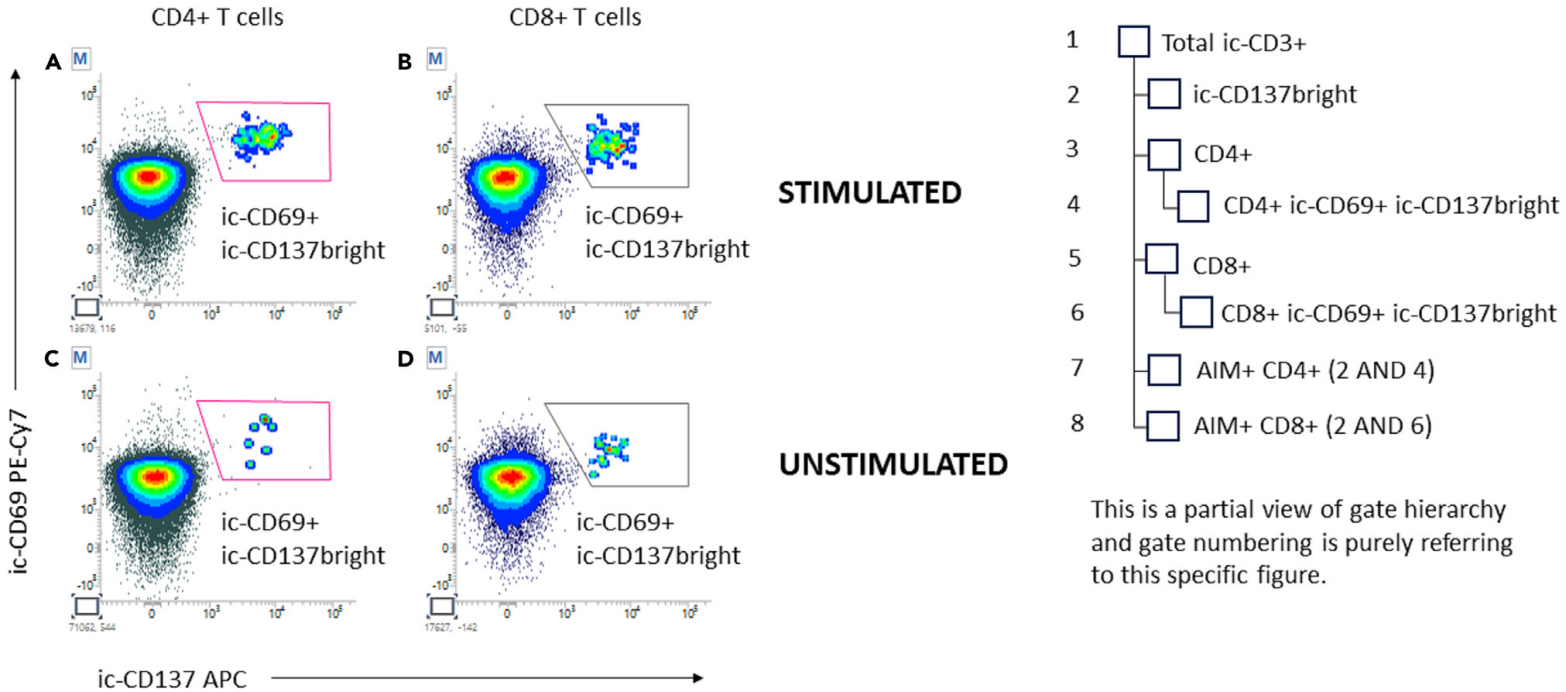
Definition of AIM+ helper and cytotoxic T lymphocytes and related gate hierarchy Plots from (A–D) display the relevant gates around the following subsets: ic-CD69+ ic-CD137bright on stimulated and unstimulated CD4+ T cells (A, C) and ic-CD69+ ic-CD137bright on stimulated and unstimulated CD8+ T cells (B, D).

**Figure 6:**
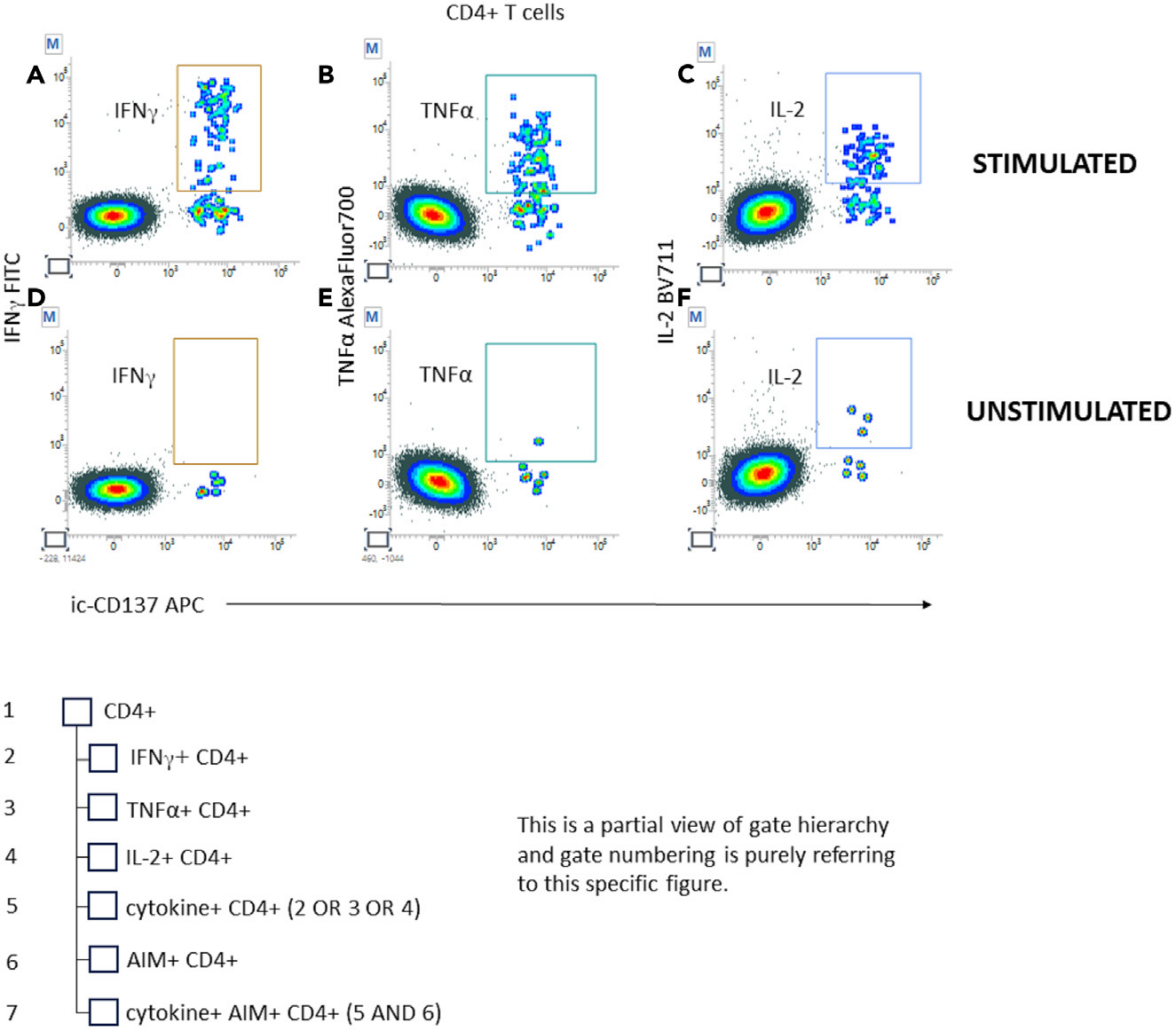
Definition of cytokine producing AIM+ helper T lymphocytes and related gate hierarchy Plots from (A–F) display the relevant gates around the following subsets: IFN-γ on stimulated and unstimulated CD4+ T cells (A, D), TNF-α on stimulated and unstimulated CD4+ T cells (B, E), and IL-2 on stimulated and unstimulated CD4+ T cells (C, F).

**Figure 7:**
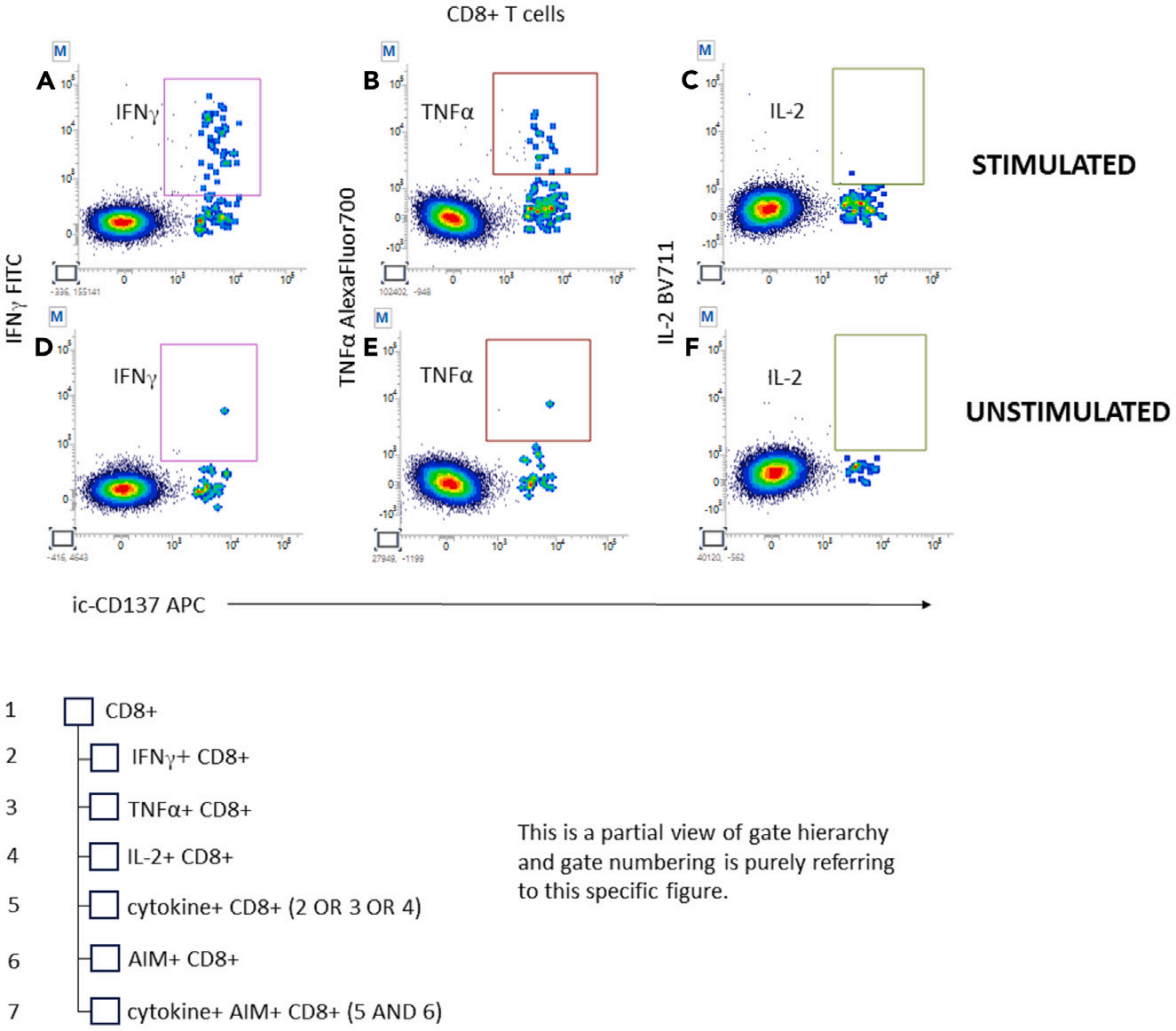
Definition of cytokine producing AIM+ cytotoxic T lymphocytes and related gate hierarchy Plots from (A–F) display the relevant gates around the following subsets: IFN-γ on stimulated and unstimulated CD8+ T cells (A, D), TNF-α on stimulated and unstimulated CD8+ T cells (B, E), and IL-2 on stimulated and unstimulated CD8+ T cells (C, F).

**Figure 8:**
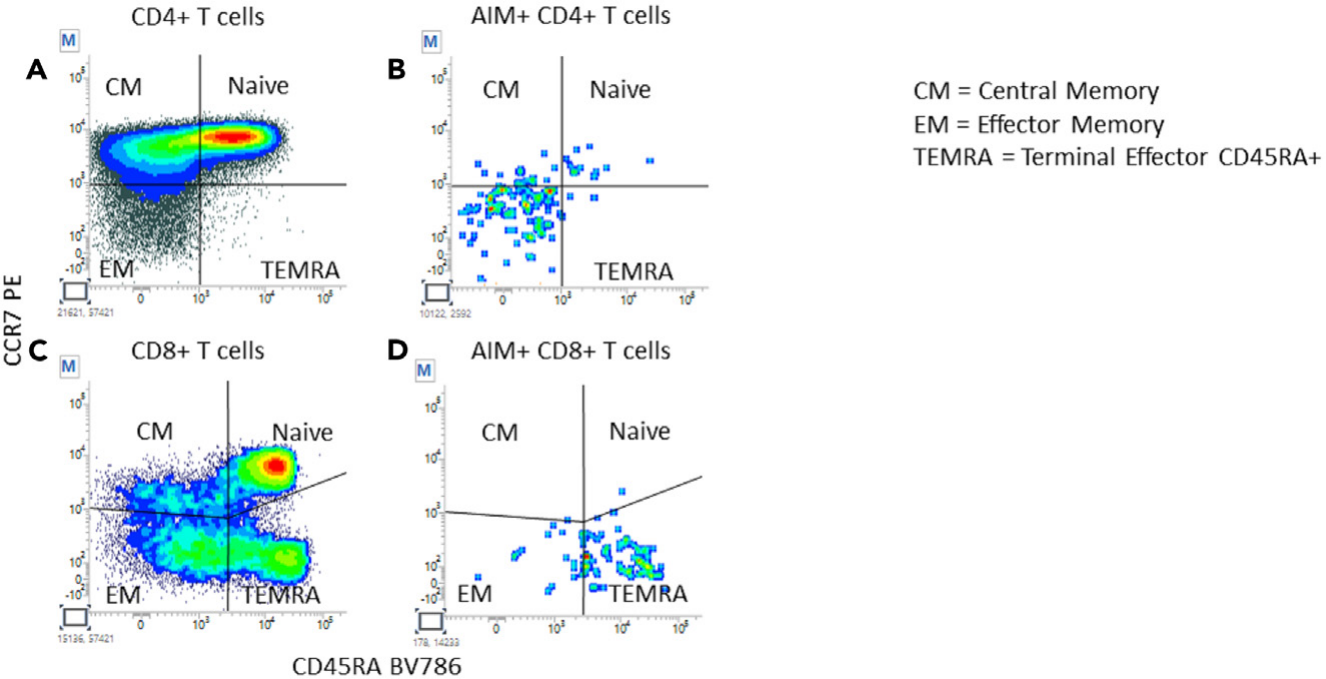
Definition of total and AIM+ helper and cytotoxic T cell maturation curves Plots from (A–D) display quadrant gates used to define the maturation curves on Total CD4+ T cells (A), AIM+ CD4+ T cells (B), Total CD8+ T cells (C), and AIM+ CD8+ T cells (D).

**Figure 9:**
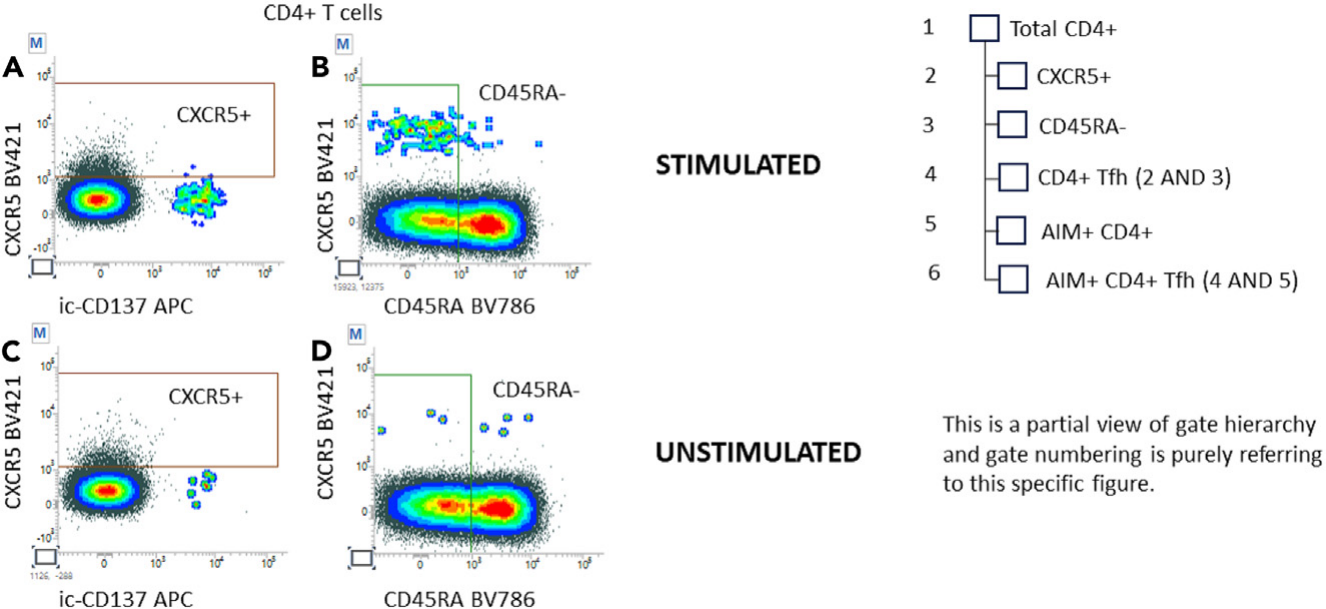
Definition of total and AIM+ T follicular helper (Tfh) CD4+ lymphocytes and related gate hierarchy Total CD4+ Follicular helper T cells have been defined as CXCR5+ (A, C) and CD45RA- (B, D) gated on stimulated and unstimulated samples, respectively. AIM+ Tfh are defined by intersecting Total CD4+ Tfh with AIM+ CD4+ T cells.

In this section, examples of analyzed data from SARS-CoV-2 reactive samples are provided, and the details of the gating strategy are explained. Figures 4, 5, 6, 7, 8, and 9 below illustrate the approach.

***Note:*** To correctly set the gate hierarchy, refer to the scheme on the lower right side of Figure 4.

Plot 4A (FSC vs. Live/Dead on All events) was used to gate live lympho-monocytes. Typically, dead cells being positive for fixable viability stain have low forward scatter and partially overlap with debris.

Subsequently plot 4B (FSC vs. SSC on Live lympho-monocytes) was created to gate lymphocytes only. Gating after dead cell exclusion will be facilitated; finally plot 4C (FSC-A vs. FSC-H on Live Lymphocytes) was used to gate singlets.

**CRITICAL:** These gates may require small adjustment between stimulated and unstimulated samples. Stimulation may in fact slightly influence scatter parameters.

Having defined singlet live lymphocytes, plot 4D (ic-CD3 vs. ic-CD137 on singlet live lymphocytes) was adopted to gate all the ic-CD3+ events. The same plot was also used to gate the ic-CD3+, ic-CD137bright cluster.

Plot 4E (CD4 vs. CD8 on ic-CD3+ T cells) was then created to gate CD4+ helper T cells and CD8+ cytotoxic T cells. Note that gating is done on Total ic-CD3+ events and not on ic-CD137bright events (Figure 4).

**CRITICAL:** ic-CD137bright gate positioning must be the same for stimulated and unstimulated samples.

***Note:*** To correctly set the gate hierarchy, refer to the scheme on the right side of Figure 5.

Activated CD4+ T cells were then gated using plots 5A and 5C (ic-CD137 vs. ic-CD69 on CD4+ T cells). “CD69+/icCD137bright” gate in plots 5A and 5C was then intersected with “ic-CD137bright” on plot 4D using “AND” logical operator to define AIM+ CD4+ T cells.

Activated CD8+ T cells were gated using plots 5B and 5D (ic-CD137 vs. ic-CD69 on CD8+ T cells). “CD69+/icCD137bright” gate in plots 5B and 5D was then intersected with “ic-CD137bright” on plot 4D using “AND” logical operator to define AIM+ CD8+ T cells (Figure 5).

**CRITICAL:** Gate positioning must be the same for stimulated and unstimulated samples. Some background in unstimulated sample is always expected.

***Note:*** To correctly set the gate hierarchy, refer to the scheme on the lower side of Figure 6.

Plots showing ic-CD137 vs. individual cytokines were created on CD4+ T cells to gate stimulated (6A, 6B, 6C) and unstimulated T helper cells (6D, 6E, 6F). Gates are capturing single cytokine+ ic-CD137bright elements.

Individual cytokine gates must be added to each other using the “OR” logical operator to define CD4+ T cell producing any of the cytokines.

Cytokine producing CD4+ T cell population is then intersected with AIM+ CD4+ population (Figure 6).

**CRITICAL:** Gate positioning must be the same for stimulated and unstimulated samples.

***Note:*** To correctly set the gate hierarchy, refer to the scheme on the lower side of Figure 7.

Plots showing ic-CD137 vs. individual cytokines were created also on CD8+ T cells to gate stimulated (7A, 7B, 7C) and unstimulated T cytotoxic cells (7D, 7E, 7F). Gates are capturing single cytokine+ ic-CD137bright elements.

Individual cytokine gates must be added to each other using the “OR” logical operator to define CD8+ T cell producing any of the cytokines.

Cytokine producing CD8+ T cell is then intersected with AIM+ CD8+ population (Figure 7).

**CRITICAL:** Gate positioning must be the same for stimulated and unstimulated samples.

To define CD4+ and CD8+ maturation curves plots 8A and 8C (CD45RA vs. CCR7 on CD4+ and CD8+ respectively) were used to place quadrant gates.

Each quadrant was intersected using the “AND” logical operator with AIM+ CD4+ or AIM+ CD8+ to generate plots 8B and 8D respectively defining AIM+ CD4+ and AIM+ CD8+ maturation curves (Figure 8).

***Note:*** AIM+ CD4+ T cells are expected to be mainly effector memory whereas AIM+ CD8+ T cells are expected to be mainly TEMRA.

Finally, plots 9A and 9C (ic-CD137 vs. CXCR5 on CD4+ T cells) were used to gate CXCR5+ events, and plots 9B and 9D (CD45RA vs. CXCR5 on CD4+ T cells) to gate CD45RA- cells.

Gates were intersected to generate the CD4+ Tfh population using “AND” logical operator.

Subsequently, the CD4+ Tfh population was intersected with the AIM+ CD4+ to generate the AIM+ CD4+ Tfh subset (Figure 9).

## Quantification and statistical analysis

In this section, data reporting through calculation of Stimulation Index is explained.

1. For both stimulated and unstimulated samples, calculate the percentages of:
  a. AIM+ CD4+ as percentage of total CD4+.
  b. Cytokine producing AIM+ CD4+ as percentage of total CD4+.
  c. AIM+ Tfh CD4+ as percentage of total CD4+.
  d. AIM+ CD8+ as percentage of total CD8+.
  e. Cytokine producing AIM+ CD8+ as percentage of total CD8+.
2. For each of the subsets, calculate the ratio between stimulated and unstimulated sample, an example is provided here below for AIM+ CD4+ T cells.

$${CD4}^{+}Stimulation\;Index=\frac{\%\;of\;{AIM}^{+}{CD4}^{+}in\;stimulated\;samples}{\%\;of\;{AIM}^{+}{CD4}^{+}\;in\;unstimulated\;samples}$$

3. Sample can be classified as responder for a given subset if both:
  a. The number of AIM+ collected events in the stimulated sample is ≥ 20. This is in fact considered the lower number of required events to detect a given subset.^10^
  b. The stimulation Index is ≥ 3.

In the 26-donor group that we analyzed for the purpose of this protocol, a single subject displayed CD4+ activation below the threshold of 20 events and two subjects displayed CD8+ activation below the threshold of 20 events; all others were above the threshold. CD4+ response (stimulation index ≥3) was detected in 20 out of 25 samples and CD8+ response (stimulation Index ≥3) was detected in 8 out of 24 samples.

## Limitations

This protocol is designed for using antigenic peptide pools. Use of whole proteins may require longer incubation time to allow processing.

The protocol uses 10^6^ PBMCs/sample which may not be enough to have more than 20 events in all cell subsets. If reporting at single donor level is required, we recommend working with a higher number of cells to get the required numbers, for any specific subset of interest (i.e., AIM+ CD4+ Tfh cells).

Correlative data on expression of other activation induced markers such as CD40L and CD25 within this protocol has not been examined and is a subject for future evaluation. Additionally, the protocol is likely to be highly adaptable to testing T-cell function in additional settings, such as immuno-oncology, by modifying the stimulating peptides, which is another area for future work.

## Troubleshooting

### Problem 1

Unwanted activation of unstimulated samples (Related to step 7 in “PBMC stimulation (Day 1)” Section).

### Potential solution

We do recommend seeding stimulated and unstimulated samples in different plates or in very separated lanes of the same plate to avoid any cross-well contamination with antigen droplets.

### Problem 2

Clog formation during sample processing (Related to step 25 in “processing of PBMCs after stimulation (Day 2)” section).

### Potential solution

Clogs may be detected upon visual inspection following cell fixation. We do recommend removing them using pipettes.

### Problem 3

**Figure 10:**
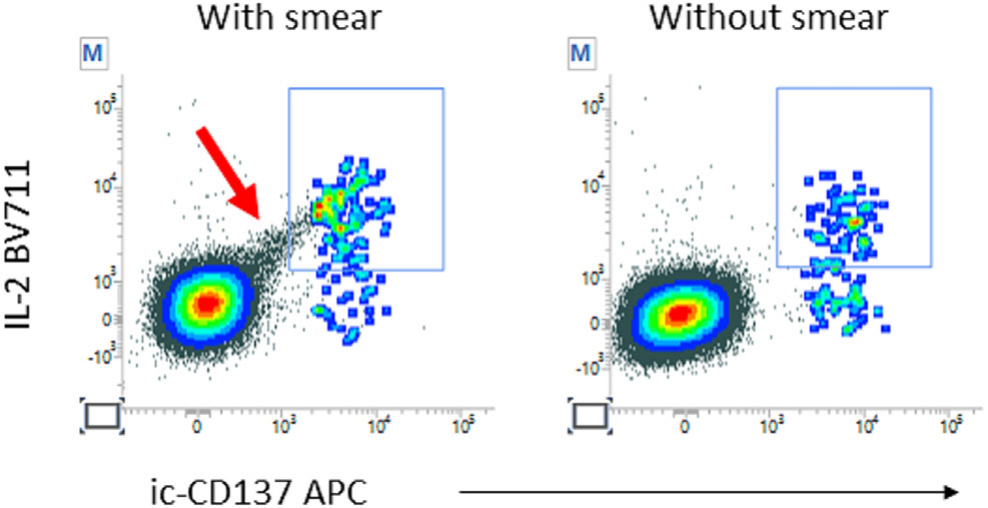
Smear generated by IL-2 BV711 antibody A smear can be randomly observed on IL-2 BV711 plots (red arrow on left plot) which can be eliminated by spinning the intracellular staining antibody cocktail (right plot).

A smear may appear on IL-2 BV711 dot plots (Related to step 26 in “processing of PBMCs after stimulation (Day 2)” Section). (Figure 10).

### Potential solution

If you happen to see smear on the IL-2 BV711 dot plot, spin down the intracellular staining antibody cocktail to eliminate the smear. Spin the cocktail down at 5,000–10,000 g (according to maximum centrifuge rotation speed) for 10 min.

### Problem 4

Clogs during flow cytometry acquisition (Related to step 34 in “processing of PBMCs after stimulation (Day 2)” section).

### Potential solution

To prevent flow cytometry clogs during acquisition, briefly run distilled water for 1 min after each sample. Normally, filtration is not required and not recommended as it may generate cell loss.

## Resource availability

### Lead contact

Further information and requests for resources and reagents should be directed to and will be fulfilled by the lead contact, Daniela Fenoglio (daniela.fenoglio@unige.it).

### Technical contact

Technical questions on executing this protocol should be directed to and will be answered by the technical contact, Gianluca Rotta (gianluca.rotta@bd.com).

### Materials availability

This study did not generate new unique reagents.

### Data and code availability

This study did not generate data set or code.

## Acknowledgments

Reagents have been provided under Material Transfer Agreement by BD Switzerland Sàrl.

## Author contributions

G.R.: conceptualization, data curation, formal analysis, and writing – original draft. V.A., S.J.B., M.D., M.F., A.G., S.H.G., I.M.R., M.M., D.J.J., J.M., V.P., J.P., B.P., H.S., C.R.M.U., and S.V.: investigation and writing – review and editing. D.F.: conceptualization and writing – review and editing.

## Declaration of interests

G.R., S.J.B., M.D., I.M.R., J.P., and S.V. are employees of Becton, Dickinson and Company.
